# Antimicrobial Activity of *Crataegi fructus* Extract Used for Potential Application in the Prevention and Treatment of Oral Diseases

**DOI:** 10.3390/medicina60010013

**Published:** 2023-12-21

**Authors:** Seoul-Hee Nam

**Affiliations:** Department of Dental Hygiene, College of Health Science, Kangwon National University, Samcheok 25945, Gangwon-do, Republic of Korea; nshee@kangwon.ac.kr; Tel.: +82-33-540-3394

**Keywords:** oral health, antimicrobial effect, *Crataegi fructus* extract, *Streptococcus mutans*, *Candida albicans*

## Abstract

*Background and Objectives*: This study evaluated the antimicrobial effect and cytotoxic potential of the natural *Crataegi fructus* extract against *Streptococcus mutans* (*S. mutans*), the causative agent of dental caries, which is a typical oral disease, and *Candida albicans* (*C. albicans*), which causes oral candidiasis. *Materials and Methods*: *Crataegi fructus* was shaken in 70% ethanol for 12 h to obtain samples through enrichment and lyophilization. Then, 1, 5, 10, 20, 30, and 40 mg/mL of the *Crataegi fructus* extract were applied to *S. mutans* and *C. albicans* to demonstrate its antimicrobial effect after 24 h. The stability of *Crataegi fructus* extract on the survival rate of human keratinocytes (HaCaT) was confirmed using water-soluble tetrazolium salt (WST-1) analysis. A one-way ANOVA determined the difference between each group. A Tukey HSD test was performed as a post-hoc analysis at the 0.05 significance level. *Results*: *Crataegi fructus* extract showed antimicrobial effects against *S. mutans* and *C. albicans* that increased in a concentration-dependent manner. However, high concentrations affected cell growth and lowered cell survival. The half maximal inhibitory concentration (IC_50_ value) of *Crataegi fructus* extract showed a survival rate of 53.04% at a concentration of 30 mg/mL, which was found to be applicable. *Conclusions*: Thus, *Crataegi fructus* extract may be used as a natural material for the prevention and treatment of oral diseases. *Crataegi fructus* demonstrates optimal antimicrobial effects without affecting cell proliferation and growth at 30 mg/mL concentration.

## 1. Introduction

Interest in oral health is gradually increasing due to reports associating oral health with improved quality of life [1]. It significantly promotes quality of life by enhancing confidence and satisfaction in social relations, as it affects not only the enjoyment of eating food, but also a person’s appearance and ability to communicate [2]. Although oral health care is important, in many cases it is neglected due to a lack of awareness of the connection between oral health and economic and social conditions [3]. Oral diseases caused by compromised oral health may lead to tooth loss; impaired masticatory function may cause a lack of nutritional intake; and pain and inflammation of the teeth and oral tissues may cause poor quality of meals, communication discomfort, and loss of confidence [4]. Moreover, reports show that people with poor oral health have poor general health [5]. Oral diseases and chronic systemic diseases have common risk factors and influence each other. Thus, they can be considered closely related [6].

Dental caries is a typical oral disease caused by bacteria, saliva, and food in dental plaque. Bacteria that cause dental caries include *Streptococcus mutans (S. mutans)* and *S. sobrinus* [7]. However, *S. mutans* is the main causative agent that adheres to tooth surfaces, and is found in large numbers in carious sites [8]. *S. mutans* resides in the oral cavity, adheres to tooth surfaces, helps secrete glucosyltransferase (GTFase), synthesizes glucan, enables bacterial adherence, and degrades sucrose. Organic acids produced by plaque bacteria lead to the dissolution of the hydroxyapatite of tooth enamel and damage the tooth surface, resulting in tooth loss [9]. Antimicrobial substances that target the causative bacterium, *S. mutans*, and inhibit its adhesion to tooth surfaces can help prevent dental caries [10].

*Candida* is detected even in most healthy people as an opportunistic pathogen. *Candida* species include *Candida albicans (C. albicans)*, *C. glabrata*, *C. krusei*, *C. parapisilosis*, and *C. tropicalis.* Among them, *C. albicans* exhibits the highest toxicity levels and growth rates [11]. *C. albicans* is usually found as a commensal microorganism, and only when there is an imbalance in the organism *C. albicans* does it begin to act as an opportunistic pathogen. It can invade the oral tissues, such as the mucous membrane and tongue, reproduce in oral epithelial cells and dentures, causing adverse effects on oral health such as discomfort, burning pain, taste abnormalities, and dysphagia during chewing [12]. Oral candidiasis may heal spontaneously due to the recovery of immunity, but most cases require antibiotic therapy [13].

Antibiotics can inhibit bacterial growth in the oral cavity and minimize the risk of oral diseases such as dental caries and oral candidiasis [14]. However, chemicals pose potential risks and many side effects when used for prolonged periods. Thus, there is constant controversy over their safety [15]. Therefore, the growing need for new antimicrobial agents has led to research on alternative products, and natural compounds isolated from plants used in traditional medicine are proposed as good alternatives to synthetic chemicals [16]. Additionally, the World Health Organization (WHO) promotes the use of traditional herbal medicines due to their low toxicity, compounds, chemical composition, and the pharmacological potential of various plant species. In recent years, many studies have focused on natural extracts that can present safe alternative options for oral disease prevention, thus avoiding the side effects of chemical agents [17].

Several studies have evaluated natural extracts for oral diseases. One study showed that *Momordica charantia* extract inhibited *S. mutans* growth and reduced its adhesion on tooth surfaces, confirming its antimicrobial efficacy against *S. mutans* [18]. Another study demonstrated that *Sambucus sieboldiana* extract inhibited the growth of *S. mutans*, confirming its antimicrobial effect and anticariogenic potential [19]. Other studies have shown the antimicrobial effects of tea tree and peppermint essential oils on *S. mutans* [20]. *Lespedeza cuneata* extract [21] and *Acanthopanax sessiliflorum* extract [22] can inhibit the growth of *C. albicans*, which suggests their usefulness as antifungal substances for preventing oral diseases. *Chamaecyparis obtuse* extract has a high level of antioxidant and antifungal activity against *C. albicans* [23].

*Crataegi fructus* is the fruit of the *Crataegus pinnatifida* Bunge tree that belongs to the *Rosaceae* family. The tree grows naturally in Korea, China, and Japan. Its fruit has a peculiar aroma and a sweet and sour taste. It is used for edible and medicinal purposes [24]. Reports show that *Crataegi fructus* is an excellent natural material for the protection of the stomach, decomposition of fat cells, enhancement of memory, and improvement of hyperlipidemia, as well as having antidepressant, antioxidant, anti-inflammatory, anticancer, and antimicrobial effects [25]. The main chemical components of *Crataegi fructus* included crude protein, crude fat, ash, and carbohydrates. The highest free sugar content was fructose, followed by glucose, mannose, ribose, and galactose. Among 16 types of amino acids, glutamic acid content was the highest, and the main fatty acids were arachidic acid, oleic acid, linoleic acid, and palmitic acid. Among the organic acids, citric acid content was the highest, and it contained vitamin C; among the minerals, it contained the highest potassium (K) content, followed by calcium (Ca), magnesium (Mg), and Ferrum (Fe) [26]. Although studies on the antimicrobial efficacy of various natural extracts in oral diseases are ongoing, there is insufficient research on the antimicrobial effect of *Crataegi fructus* against oral microorganisms that cause oral diseases. 

The epidermis acts as a barrier to protect the body from external substances, and HaCaT cells (human keratinocytes) that make up the oral epidermis are used as in vitro models to evaluate cytotoxicity [27,28,29]. Evaluating the cellular stability of promising plant extract oral antimicrobial agents is crucial for developing effective and safe agents. However, there are no studies on the cell stability of *Crataegi fructus* extracts to evaluate safe oral application. 

Therefore, in this study, *Crataegi fructus* extract was applied to *S. mutans*, a typical bacterium that induces dental caries, to confirm its anti-caries effect, and *C. albicans*, the cause of oral candidiasis, to assess its antifungal effect. Moreover, the study evaluated the cytotoxicity of *Crataegi fructus* extract to confirm its usefulness for safe treatment or prevention of oral diseases.

## 2. Materials and Methods

### 2.1. Crataegi fructus Extract

The *Crataegi fructus* used in this study was grown in China and purchased from Cheongmyeong Co., Ltd. (Chungju, Gyeongsangbuk, Republic of Korea). Five times the amount of 70% ethanol was added to 100 g of pulverized *Crataegi fructus* and shaken at 60 °C for 24 h. The ratio of extract to solvent is 1:5. After filtering the extract three times using filter paper (Advantec No. 2, Tokyo, Japan), the supernatant that passed through the filter paper was concentrated using a rotary vacuum evaporative concentrator (N-1300E.V.S. EYELA Co., Tokyo, Japan). The concentrated extract was freeze-dried with a freeze dryer (FD5508, Ilshin Lab, Yangju, Kyunggi-do, Republic of Korea) to obtain the powder. The obtained powder was stored at −20 °C until use and diluted in dimethyl sulfoxide (DMSO, Sigma-Aldrich, St. Louis, MO, USA). 

### 2.2. Antimicrobial and Antifungal Activity 

Strains of *S. mutans* (KCTC 3065/ATCC 25175) and *C. albicans* (KCTC 7965/ATCC 10231) were used after subculture in a brain-heart infusion broth (BHI broth, Sigma-Aldrich, St. Louis, MO, USA) and a yeast mold broth (YM broth, Difco, Foley, AL, USA), respectively, at 37 °C for 24 h. The colony-forming units (CFU)/mL were measured to determine the number of viable cells in the *Crataegi fructus* extract against *S. mutans* and *C. albicans*. After diluting the extract to concentrations of 1, 3, 5, 10, 20, 30, and 40 mg/mL in BHI broth and YM broth, 1 × 10^5^ *S. mutans* and 1 × 10^4^ *C. albicans* were mixed and subjected to culture at 37 °C for 24 h, then spread on a BHI agar medium and a YM agar medium, incubated for 24 h, and tested for CFU/mL.

### 2.3. Minimal Inhibitory Concentration (MIC) and Minimal Bactericidal Concentration (MBC) or Minimal Fungicidal Concentration (MFC) Measurements

The bacterial concentrations used in this measurement were about 1 × 10^5^ *S. mutans* and 1 × 10^4^ *C. albicans* CFU/mL, and the concentration gradients of *Crataegi fructus* extract were 1, 3, 5, 10, 20, 30, and 40 mg/mL. An inoculum suspension was incubated at 37 °C for 24 h. MIC and MBC or MFC values were determined by spreading different concentrations (1, 3, 5, 10, 20, 30, and 40 mg/mL) from each mixed tube onto the plate. MIC was set as the lowest concentration that inhibited the growth of a given strain of bacteria. The MBC or MFC was a concentration that kills 99.9% of bacteria and has been described as the lowest extract concentration at which no bacterial growth was observed. All tests were performed in triplicate.

### 2.4. Cytotoxic Effects

HaCaT cell lines (human keratinocytes) were grown in recommended culture media supplemented with Dulbecco’s Modified Eagle’s Medium (DMEM) (USA) containing 10% (*v*/*v*) heat-inactivated fetal bovine serum (FBS, Gibco, Grand Island, NY, USA) and 1% penicillin/streptomycin (100 μL/mL, Gibco, Grand Island, NY, USA) in a 5% CO_2_ humidified atmosphere at 37 °C. The cells were plated in a 96-well culture plate containing a final volume of 100 μL of medium. A suspension of 1  ×  10^5^ cells/cm^2^ was seeded in 96-well plates and incubated at 37 °C for 24 h.

An evaluation of cytotoxicity was performed using water-soluble tetrazolium salt-1 (WST-1) analysis. The medium was changed to serum-free DMEM containing 1, 3, 5, 10, 20, 30, and 40 mg/mL concentrations of *Crataegi fructus* extract. After incubation for 3 h, the incubation medium was carefully aspirated, and the WST-1 solution was added to each well. The plates were incubated at 37 °C for 2 h, and the absorbance data were detected using a microplate reader (Multiskan FC, Thermo Fisher Scientific, Waltham, MA, USA) at 490 nm. Additionally, each test was replicated three times independently. 

### 2.5. Statistical Analysis

Statistical analysis of the antimicrobial and antifungal effect was performed using IBM SPSS Statistics 24.0 (SPSS Inc., Chicago, IL, USA) to analyze the results of different concentrations of the *Crataegi fructus* extract at the 95% significance level, using one-way ANOVA. A Tukey HSD test was performed as a post-hoc test to confirm the differences according to the concentration of the extract. 

## 3. Results

### 3.1. Changes in Oral Disease-Causing Bacteria and Yeast

**Figure 1:**
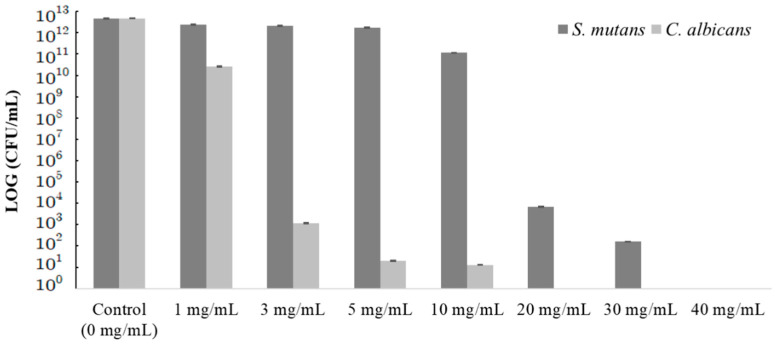
Antimicrobial effect of *Crataegi fructus* extract on *S. mutans* and *C. albicans*.

The antimicrobial and antifungal effect against *S. mutans* and *C. albicans* was evident as the concentration of the *Crataegi fructus* extract increased (Figure 1). Growth inhibition confirmed a decrease in the CFU/mL with increased concentrations of *Crataegi fructus* extract, indicating a clear killing effect on *S. mutans* and *C. albicans*. 

Table 1 presents statistical changes in CFU/mL. Statistically significant differences are shown between *S. mutans* and *C. albicans* treated with different concentrations of the *Crataegi fructus* extract (*p* < 0.05). The antimicrobial effect of *Crataegi fructus* extract added to the *S. mutans* increased with higher concentrations, although there was no statistically significant difference between the numbers of CFU/mL of *S. mutans* between groups when treated with 20 mg/mL and 40 mg/mL of the *Crataegi fructus* extract. As for *C. albicans*, there was also a significant difference between the number of CFU/mL with increasing concentration of *Crataegi fructus* extract. There was a clear decrease in CFU/mL depending on the application of *Crataegi fructus* extract, but there was no statistically significant difference from 3 mg/mL to 40 mg/mL (Table 1).

### 3.2. Determination of MIC and MBC or MFC

**Figure 2:**
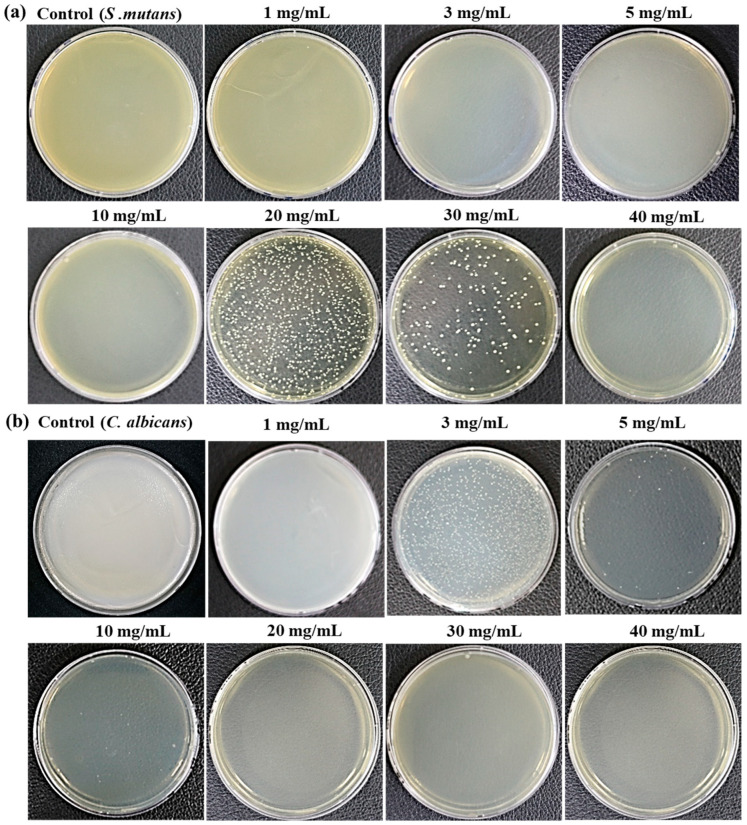
The MIC and MBC or MFC against (**a**) *S. mutans* and (**b**) *C. albicans* using *Crataegi fructus* extract after 24 h.

The MIC of *Crataegi fructus* extract was 30 mg/mL for *S. mutans* and 10 mg/mL for *C. albicans*. The MBC and MFC values showed that *S. mutans* was completely killed by 40 mg/mL, and *C. albicans* was completely killed by 20 mg/mL of extract application (Figure 2).

### 3.3. Cell Viability Assay Using HaCaT Cells

**Figure 3:**
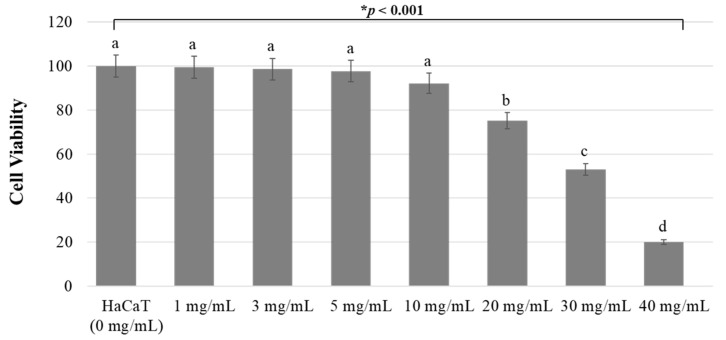
The cytotoxicity elicited by *Crataegi fructus* extract on HaCaT cells after 3 h of treatment. * Different letters (a, b, c, and d) indicate statistically significant results of the one-way ANOVA and post-hoc Tukey HSD (*p* < 0.05).

Cell viability was evaluated by a WST-1 assay for toxicity detection, as shown in Figure 3. *Crataegi fructus* extract significantly inhibited the viability of HaCaT cells in a concentration-dependent manner (*p* < 0.05). Treatment with *Crataegi fructus* extract induced a reduction in cell viability at approximately 99.44%, 98.65%, 97.72%, 92.23%, 75.17%, 53.04%, and 20.05% after applying 1, 3, 5, 10, 20, 30, and 40 mg/mL for 3 h. A 30 mg/mL concentration of the *Crataegi fructus* extract resulted in a 50% reduction of cell viability and half maximal inhibitory concentration (IC_50_ value).

## 4. Discussion

Health is the most fundamental right of all citizens, and is crucial to human life [30]. Oral care is essential, not only for maintaining healthy teeth and oral tissues, but also for improving physical health [31]. Moreover, since oral health is closely related to social relations and overall quality of life, oral health care is also important to improve quality of life [32]. Thus, tooth loss resulting from oral health problems can adversely affect general health and quality of life [33]. 

The oral cavity is an open ecosystem that receives a constant influx of microorganisms from the food we eat and the air we breathe, providing environmental conditions suitable for bacterial growth and reproduction. In general, the mucous membranes of the lips, palate, cheeks, tongue, gums, teeth, and saliva are densely populated by complex microbial communities, providing a favorable environment for the growth and reproduction of various microorganisms [34]. The bacterial flora is stable in a healthy state, but pathogenic flora resulting from an increase or decrease in the number of specific bacteria produce oral bacterial films, causing bad breath, dental caries, gingivitis, and periodontitis [35]. Poor oral hygiene, smoking or tobacco chewing, inadequate nutrition, and overuse of antibiotics can disrupt the homeostasis between the oral microbial communities and may even lead to antimicrobial resistance [36]. Therefore, infections and antibiotic resistance caused by oral diseases remain important health problems worldwide, causing serious diseases. To maintain healthier oral conditions, targeting and managing harmful oral disease-producing bacteria will help maintain good health and well-being.

Tooth brushing is the most basic and effective way to reduce oral bacteria. Furthermore, mechanical and chemical oral hygiene management methods can reduce oral bacterial load and improve overall oral health [37]. Currently, oral products such as toothpaste and chemical mouthwash agents are widely used to maintain oral health due to their strong antimicrobial effect. However, they cause problems and side effects, such as tooth discoloration, damage to the oral mucosa, and antibiotic resistance [38]. Moreover, reports show that the ethanol component of mouthwash may contribute to the development of oral cancer [39]. With reports on cell toxicity of various chemical agents, studies are focusing on pharmaceutically active natural extracts that are safe and devoid of side effects. These natural extracts are preferred due to their pharmacological efficacy and minimal side effects [40]. 

*Crataegi fructus* extract is used in Oriental medicine for promoting digestion and gastrointestinal function, improving blood circulation, and removing stagnant blood [41]. *Crataegi fructus* extract contains catechin (the main active ingredient), a flavonoid in many plants, exhibiting several pharmacological effects [42]. Therefore, in this study, *Crataegi fructus* extract, found to have anti-inflammatory and antimicrobial effects, was applied to *S. mutans* (a cariogenic bacterium) and *C. albicans* (causing oral candidiasis) to examine its antimicrobial effect on oral diseases. 

The *Crataegi fructus* extract was added to each strain at concentrations of 1, 3, 5, 10, 20, 30, and 40 mg/mL, subjected to culture for 24 h, and tested for CFU/mL of the strains. The CFU/mL of *S. mutans* and *C. albicans* decreased with increasing concentrations of the *Crataegi fructus* extract. The inhibitory effect of *S. mutans* (4.58 ± 1.0 × 10^12^) growth according to the concentration of the *Crataegi fructus* extract was 2.37 ± 2.1 × 10^12^ at 1 mg/mL, 2.12 ± 1.6 × 10^12^ at 3 mg/mL, 1.75 ± 1.9 × 10^12^ at 5 mg/mL, 1.14 ± 1.7 × 10^11^ at 10 mg/mL, 6.79 ± 2.7 × 10^3^ at 20 mg/mL, 1.60 ± 1.3 × 10^2^ at 30 mg/mL, and 0.00 at 40 mg/mL. The inhibitory effect of *C. albicans* (4.75 ± 1.0 × 10^12^) growth was 2.59 ± 1.7 × 10^10^ at 1 mg/mL, 1.16 ± 1.9 × 10^3^ at 3 mg/mL, 2.00 ± 2.1 × 10^1^ at 5 mg/mL, 1.30 ± 1.4 × 10^1^ at 10 mg/mL, and 0.00 at 20–40 mg/mL. These results indicate that higher concentrations of *Crataegi fructus* extract exhibited greater antimicrobial efficacy against oral disease-producing bacteria and yeast. Results showed that hawthorn fruit extract exhibited more effective antimicrobial activity against *C. albicans* than against *S. mutans*. In particular, *C. albicans* showed an antimicrobial and antifungal effect of 99.9% at a low concentration of 3 mg/mL of *Crataegi fructus* extract. 

These results are similar to those of a study that first demonstrated the antimicrobial effect of *citrus peel* extract against *S. mutans* at a concentration of 5 mg/mL, which gradually increased as the concentration of the extract increased to 10 and 20 mg/mL [43]. *Citrus sinensis peel* extracts inhibited microbial growth at very high concentrations of 32 mg/mL against *S. mutans* [44]. In another study, following *Solanum nigrum* extract application to *C. albicans* at concentrations of 5, 10, 20, and 40 mg/mL, the number of CFU/mL of *C. albicans* decreased starting at 5 mg/mL and kept decreasing as the concentration of the extract increased [45]. The MIC of the *Pechuel-Loeschea leubnitziae* leaf extract for *C. albicans* was observed at 4.688 mg/mL, and the bacteriolytic effect was found at 37.5 mg/mL [46]. In this study, for *S. mutans*, the antimicrobial effect began to appear from 1 mg/mL, with no significant difference between 20 and 40 mg/mL, and the complete bacteria-killing effect was seen at 40 mg/mL. For *C. albicans*, the antimicrobial effect also began to appear from 1 mg/mL, but there was no distinct difference according to the concentration at 3 and 40 mg/mL, and the complete bacteria-killing effect was seen at 40 mg/mL. *Crataegi fructus* extract was more potent and effective against oral pathogens. Thus, the *Crataegi fructus* extract showed an antimicrobial effect against *S. mutans* and *C. albicans* starting from 1 mg/mL, but it exhibited a stronger antimicrobial effect against *C. albicans*, indicating stronger antifungal efficacy against oral candidiasis.

Herbal medicines may be considered safe because they are natural, but they can be toxic if not used correctly [47]. In vitro methods of cytotoxicity testing should quantify cell viability and growth [48]. Therefore, this study evaluated the effect of *Crataegi fructus* extract on cell proliferation of HaCaT cell lines. Cell growth began to decrease at a concentration of 20 mg/mL of *Crataegi fructus* extract; thus, 40 mg/mL of *Crataegi fructus* extract application was not safe for oral epidermal cells, and the IC_50_ value was 30 mg/mL. *Crataegi fructus* extract reduced HaCaT cell proliferation in a dose-dependent manner. Doses of 100 mg/mL of *Desmodium adscendens* extract are not safe and should be used with caution. Reports show that appropriate doses of 1 mg/mL and 10 mg/mL are safe and have a cytoprotective effect [49]. In this study, higher concentrations ranging from 1 mg/mL to 30 mg/mL were found to be appropriate and safe. These results demonstrated that up to 30 mg/mL of *Crataegi fructus* extract is safe for HaCaT cells and has excellent anti-caries and antifungal effects. Expanding the scope of applying functional natural materials is necessary for developing and using oral hygiene care products. Therefore, more research on natural substances is desirable to provide a promising source of natural products for improving oral health. 

A limitation of this study is that we did not conduct analytical methodologies, including extraction, isolation, and characterization of active ingredients, which are critical for utilizing biologically active compounds from natural extracts for medicinal use. *Crataegi fructus* extract can be used in oral hygiene products with antimicrobial properties for minimizing the risk of dental caries and oral candidiasis. Additional studies are needed to isolate pure compounds associated with antimicrobial activity. Additionally, in vivo testing is needed to evaluate its toxicity and potential against oral pathogens. *Crataegi fructus* extract has antimicrobial efficacy, and human clinical studies are necessary to confirm changes in the oral environment following clinical application at safe concentrations.

## 5. Conclusions

*Crataegi fructus* extract, when applied to *S. mutans* and *C. albicans* for preventing and treating oral diseases, exhibited antimicrobial and antifungal efficacy that increased with increasing concentration. The study showed excellent antimicrobial effects when the *Crataegi fructus* extract was applied at a concentration of 40 mg/mL for *S. mutans* and 20 mg/mL for *C. albicans*. However, *Crataegi fructus* extract of up to 30 mg/mL was found to exhibit effective antimicrobial activity, and is believed to be an effective natural extract for the prevention and treatment of oral diseases at a concentration that does not affect cell growth or cause cytotoxicity. 

## Figures and Tables

**Table 1:** Comparison of CFU/mL and different concentrations of *Crataegi fructus* extract. Unit: Mean ± S.D.

Group	0 mg/mL	1 mg/mL	3 mg/mL	5 mg/mL	10 mg/mL	20 mg/mL	30 mg/mL	40 mg/mL	ANOVA *p*-Value
*S. mutans*	4.58 ± 1.0 10^12,a^	2.37 ± 2.1 10^12,b^	2.12 ± 1.6 10^12,b^	1.75 ± 1.9 10^12,c^	1.14 ± 1.7 10^11,d^	6.79 ± 2.7 10^3,e^	1.60 ± 1.3 10^2,e^	0.00 ^e^	0.000 *
*C. albicans*	4.75 ± 1.0 10^12,a^	2.59 ± 1.7 10^10,b^	1.16 ± 1.9 10^3,c^	2.00 ± 2.1 10^1,c^	1.30 ± 1.4 10^1,c^	0.00 ^c^	0.00 ^c^	0.00 ^c^	0.000 *

* Different letters (^a^, ^b^, ^c^, ^d^, and ^e^) indicate statistically significant results of the one-way ANOVA and post-hoc Tukey HSD (*p* < 0.05).

## Data Availability

The data presented in this study are available upon request from the corresponding author.
